# Obesity-related glomerulopathy, A growing kidney burden in the obesity pandemic

**DOI:** 10.1007/s10157-025-02804-7

**Published:** 2025-12-18

**Authors:** Mahtab Mashayekhi, Jonathan E. Zuckerman, Sahar H. Koubar, Junnan Wu, Jianbo Qing, Amir Abdipour, Edgar Lerma, Warren Peters, Sayna Norouzi

**Affiliations:** 1 Division of Nephrology, Department of Medicine, Loma Linda University Medical Center, Loma Linda, CA USA; 2 Department of Pathology and Laboratory Medicine, UCLA David Geffen School of Medicine, Los Angeles, CA USA; 3 Division of Nephrology and Hypertension, University of Minnesota, Minneapolis, MN USA; 4 Department of Nephrology, Sir Run Run Shaw Hospital, Zhejiang University School of Medicine, Hangzhou, 310000 China; 5 Department of Medicine, Section of Nephrology, Advocate Christ Medical Center, Oak Lawn, IL USA; 6 Obesity Research, Loma Linda University School of Public Health, Loma Linda, USA

**Keywords:** Obesity, Related Glomerulopathy, ORG, Obesity, Related FSGS, GLP1 receptor agonists

## Abstract

Obesity can cause the progression of kidney disease through hemodynamic, structural, and metabolic changes, and predispose individuals to arterio-nephrosclerosis, diabetic nephropathy, and focal segmental glomerulosclerosis (FSGS), leading to chronic kidney disease (CKD). Obesity-Related Glomerulopathy (ORG) is defined as clinical obesity and biopsy-proven glomerulomegaly with or without the existence of FSGS. However, pathologic changes of ORG are not pathognomonic or specific. Glomerular hypertrophy, maladaptive segmental glomerulosclerosis, as well as in some cases diabetic-like changes may be seen secondary to any cause of acquired or congenital reduced nephron mass with compensatory hypertrophy as well as glomerular hypoxia. This review aims to provide a comprehensive overview of the mechanisms causing ORG and explore current diagnostic challenges and therapeutic strategies, emphasizing the role of weight management and emerging targeted therapies.

## Introduction

The alarming increase in the worldwide prevalence of obesity raises major concerns about obesity-related complications such as diabetes mellitus (DM), cardiovascular disease, and chronic kidney disease (CKD) [1]. The World Health Organization (WHO) describes a body-mass-index (BMI) > 25 kg/m^2^ as overweight and BMI > 30 kg/m^2^ as obesity [2].

Based on the Global Burden of Disease data, an estimated 2.11(95% UI 2·09–2·13) billion adults aged ≥ 25 years worldwide were living with overweight or obesity in 2021, with a prevalence ~ 45.1% [44.7–45.4]. Global forecasts indicate that by 2050, the number of adults with overweight or obesity will increase to about 3.80 billion (95% UI 3.39–4.04), corresponding to over 60% of the projected adult population at that time [3].

Obesity can cause the progression of kidney disease through hemodynamic, structural, and metabolic changes. It increases the risk of conditions such as arterio-nephrosclerosis, diabetic nephropathy, and focal segmental glomerulosclerosis (FSGS), which lead to CKD [4]. Obesity-related CKD independent of diabetes and hypertension, involves chronic inflammation, and lipotoxicity. Excess adipose tissue promotes kidney fibrosis and podocyte injury by releasing inflammatory cytokines and oxidative stress, which combined with glomerular hyperfiltration can cause secondary FSGS, mostly in the Perihilar region of glomeruli [5, 6].

Obesity-Related Glomerulopathy (ORG) is defined as obesity, BMI ≥ 30 kg/m^2^ in Western populations or ≥ 25 kg/m^2^ in Asian populations, and biopsy-proven glomerulomegaly with or without co-existing FSGS lesions [7]. Obesity-related FSGS (O-FSGS) is a secondary form of FSGS that arises due to obesity-induced glomerular stress and podocyte injury, which is characterized by sub-nephrotic or nephrotic range of proteinuria (> 3.5 g/day) without full nephrotic syndrome, progressive kidney dysfunction, and distinct histopathological features, including glomerular hypertrophy (termed glomerulomegaly) and perihilar-predominant segmental sclerosis. It is different from primary FSGS also by its lower incidence of nephrotic syndrome, milder podocyte injury, and a more indolent disease course [8]. There is a significant increase in the prevalence of FSGS lesions among obese patients undergoing kidney biopsy, in parallel with the rise in obesity over recent decades. Obese individuals with ORG and additional kidney diseases had a significantly greater risk of progressing to end-stage kidney disease (ESKD) compared to those with ORG alone (RR 2.48; 95% CI, 1.09–5.64) [9]. While bariatric surgery was one of the most effective therapies for ORG in the past, the advancement of new therapies, such as Glucagon-like peptide-1 receptor agonists (GLP-1RAs), brings renewed optimism for effective and non-invasive management options for ORG [7].

In this review, we aim to provide a comprehensive overview of the mechanisms causing ORG (and O-FSGS), including obesity-induced glomerular stress, podocyte injury, and maladaptive kidney changes. We explore current diagnostic challenges and therapeutic strategies, emphasizing the role of weight management and emerging therapies.

### Pathophysiology of ORG

**Fig. 1:**
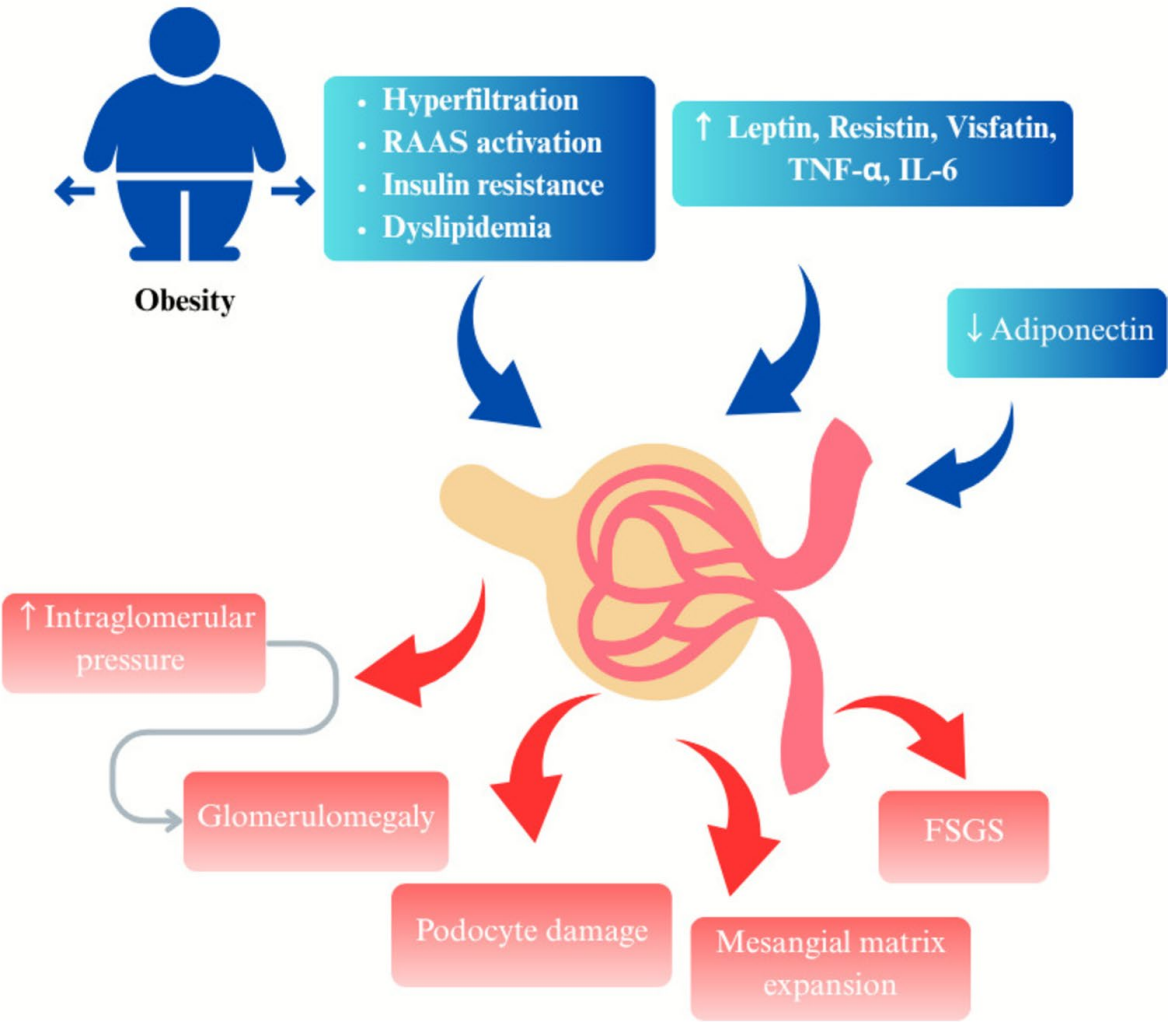
Pathophysiology of obesity related glomerulopathy

Obesity-induced hemodynamic changes cause an increase in renal plasma flow (RPF) and glomerular filtration rate (GFR), with the increase in GFR being greater than the increase in RPF, indicating selective afferent-arteriole vasodilation leading to glomerular hyperfiltration. This results in an increase in intraglomerular pressure, contributing to glomerulomegaly and podocyte stress. Furthermore, sodium retention due to increased activity of sodium-glucose cotransporters (SGLT1 and SGLT2) in the proximal tubule reduces tubuloglomerular-feedback activation, exacerbating hyperfiltration. The renin–angiotensin–aldosterone system (RAAS) also plays an important role, as angiotensin II (AngII) preferentially constricts efferent arterioles, elevating transcapillary hydraulic pressure and sustaining hyperfiltration. Moreover, aldosterone mediated oxidative stress and fibrosis lead to podocyte injury, further aggravating kidney dysfunction (Fig. 1) [10, 11]. In a recent study on 200 patients with obesity and 200 potential living kidney transplant donors as a comparison group, findings revealed creatinine clearance was higher in obese patients than potential transplant donors, but this difference was underestimated when adjusted for body surface area (BSA). Proteinuria levels in obese patients were greater than potential kidney donors. Regression analysis indicated a strong association between creatinine clearance and proteinuria in obese patients and highlights that hyperfiltration and excessive proteinuria are common in obesity [12].

**Fig. 2:**
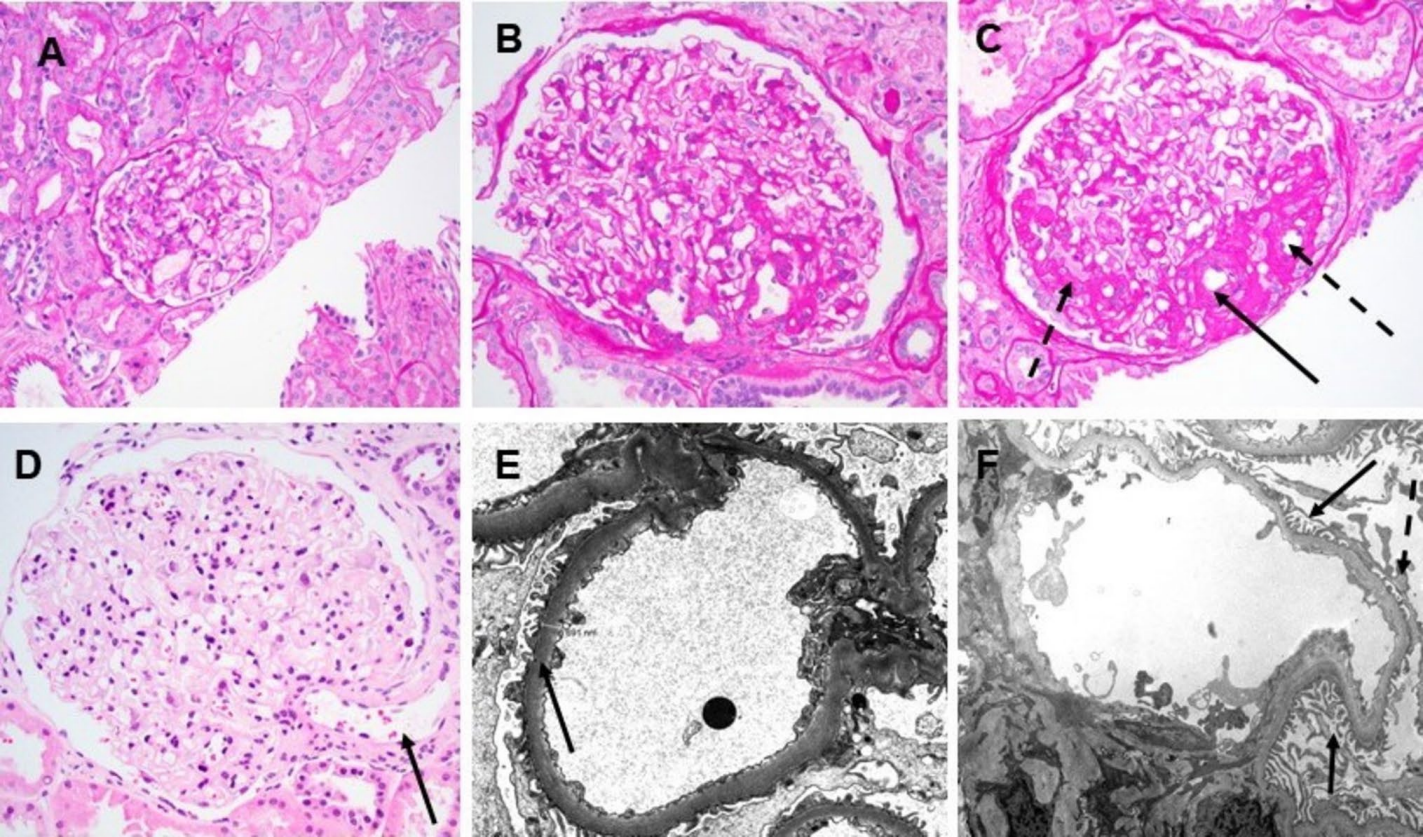
Morphologic features commonly observed with obesity related glomerulopathy (ORG). **A** Normal appearing glomerulus from a patient with a normal body mass index (19.5; adult minimal change disease patient). **B** A hypertrophied glomerulus from an obese patient (body mass index 32) taken at the same magnification as panel A. **C** Peri-hilar type segmental glomerulosclerosis in an enlarged glomerulus from an obese patient; solid arrow points to the glomerular hilum, dotted arrows point to associated glomerular capillary tuft segments involved by segmental sclerosis. **D** An enlarged glomerulus from an obese patient demonstrating mild hilar arterial dilation suggestive of glomerular hyperfiltration (solid arrow). Electron micrographs from patients with ORG demonstrating mildly thickened glomerular basement membranes (**E**, solid arrows) and mild segmental podocyte food process effacement (**F**, solid arrows point to intact foot processes, dashed arrow points to an area of foot process effacement) **A-C** Periodic acid Schiff stain, original magnification 200x; **D** Hematoxylin and eosin stain, original magnification 200x; E original magnification 4800x, F original magnification 2900x)

The persistent hyperfiltration caused by obesity results in glomerulomegaly, and increased capillary flow and filtration lead to mesangial cell proliferation and extracellular matrix expansion and stretching of the glomerular capillary loops and glomerular basement membrane. These changes cause excessive stress on podocytes. As podocytes undergo mechanical strain, filtration integrity is compromised resulting in proteinuria. Chronic stress and inflammatory mediators, such as leptin and AngII, further promote podocyte detachment, resulting in podocytopenia, which diminishes the ability of the glomerulus to maintain structural homeostasis. Moreover, mesangial expansion can also occur due to prolonged hyperfiltration and metabolic disturbances further reducing filtration efficiency. The loss of podocytes and sustained mechanical stress drive segmental glomerulosclerosis (often peri-hilar variant) and ultimately global glomerulosclerosis**,** resulting in kidney dysfunction, lower eGFR, and increases the risk of CKD progression (Fig. 2) [4, 13].

Glomerular hypertrophy does not directly correlate with proteinuria or glomerulosclerosis, it triggers stress on podocytes, contributing to their injury and loss, although it may be the only histologic abnormality in obese patients with proteinuria. A significant reduction in podocyte number is strongly associated with the rise in proteinuria and kidney function decline, highlighting podocyte depletion as the most significant factor in O-FSGS development. Mechanical stress, which happens especially in the perihilar area where filtration pressure is highest, is caused by pathways involving AngII and Transforming Growth Factor-Beta (TGF-β), both of which promote oxidative stress, disrupt slit diaphragm proteins, reduce nephrin levels, and weaken podocyte adhesion to the basement membrane. AngII also changes the glomerular basement membrane by stimulating collagen production and increasing VEGF, further compromising podocyte integrity [8].

A systematic review and meta-analysis showed that every 1 kg/m^2^ rise in BMI, increases the risk of low eGFR (below 60 mL/min/1.73 m^2^) by approximately 2% [14].

Obesity leads to insulin resistance and negatively impacts kidney function as proinflammatory cytokines like tumor necrosis factor-α (TNF-α) and interleukin-6 (IL-6) contribute to kidney damage [6]. The distribution of adipose tissue may influence the risk and progression of ORG. Visceral adiposity, rather than subcutaneous fat accumulation, is more strongly associated with kidney dysfunction due to its higher metabolic activity and secretion of proinflammatory cytokines. These mediators promote glomerular hyperfiltration and endothelial dysfunction, thereby accelerating kidney injury. In contrast, subcutaneous adiposity has been shown to have less impact on renal hemodynamics [15, 16]. Imaging-based indices such as visceral-to-subcutaneous fat ratio and waist-to-hip ratio have been shown to correlate more closely with kidney outcomes than BMI alone, which highlights the importance of body-fat distribution in ORG risk assessment [17, 18].

Leptin, a type of adipokine, is elevated in obese patients, and contributes to kidney damage by promoting glomerulosclerosis, interstitial fibrosis, and inflammation. It activates the Janus kinase-signal transducer and activator of transcription (JAK2-STAT) signaling pathway in kidney mesangial cells, leading to increased TGF-β1 production, and stimulates the deposition of extracellular matrix proteins like type IV collagen and fibronectin, ultimately resulting in glomerular scarring. Leptin contributes to endothelial dysfunction by increasing the expression of adhesion molecules like vascular cell adhesion molecule-1 (VCAM-1) and intercellular adhesion molecule-1 (ICAM-1), leading to vascular remodeling and kidney inflammation [6].

Adiponectin is another adipokine that has receptors on glomerular cells, including podocytes, suggesting potential direct effects within the kidney, such as antifibrotic effects. Serum adiponectin levels are typically reduced in obesity [19]. A study by Sharma et al. demonstrated that lower plasma adiponectin levels were closely associated with increased albuminuria in obese African American individuals without diabetes or existing kidney disease. In adiponectin-deficient mice, the absence of adiponectin led to greater albuminuria, oxidative stress, and podocyte foot process effacement. Treatment of these mice with adiponectin caused normalization in albuminuria, improved podocyte foot process effacement, and reduced oxidative stress [20]. Visfatin, another type of adiponectin associated with obesity, can be synthesized by renal glomerular mesangial cells. It increases profibrotic molecules such as TGF-β1, plasminogen activator inhibitor-1 (PAI-1), and type-I collagen [21]. As such, visfatin may have role in development of O-FSGS.

Resistin, a proinflammatory mediator, is elevated in obesity and CKD, and may be linked to ORG. It promotes insulin resistance and cytokine-release such as TNF-α, IL-6, and monocyte chemoattractant-protein-1 (MCP-1), activating macrophages in a proinflammatory response, with subsequent glomerulosclerosis and tubulointerstitial fibrosis [22].

Alterations in gut microbiota and intestinal barrier dysfunction are critical factors in the progression of kidney disease [23]. Obesity is closely linked to changes in the gut microbiota and intestinal dysbiosis, which increase gut permeability, allowing inflammatory mediators to enter the bloodstream, leading to systemic inflammation and metabolic disturbances that can exacerbate kidney dysfunction [24]. A study investigating the therapeutic effects of cyanidin-3-glucoside (C3G) in a mouse model ORG, showed promising results. After eight weeks of C3G treatment, significant improvements were observed in metabolic, biochemical, and histological markers of kidney injury in ORG mice. These included reductions in proteinuria, lipid levels, and tubular damage. Fecal 16S rRNA sequencing revealed that ORG mice exhibited lower gut microbiota diversity compared to control mice, suggesting an altered gut microbiota composition in ORG. Multi-omics analyses further revealed that C3G treatment modulated glycerophospholipid metabolism, enhanced gut microbiota diversity, and inhibited the PPARγ/CD36 pathway in proximal tubular cells. This pathway inhibition was associated with a reduction in lipid droplet deposition in the kidney [25]. It is worth noting that while some recent studies have provided evidence for the relationship between gut microbiota and ORG, most human studies are focused on correlations. Although some studies have validated causal relationships using animal models, the causal links between gut microbiota and ORG remain unclear in the majority of cases [26]. This requires further in-depth clinical trials and longitudinal studies, including targeted interventions such as gut bacteria or their metabolites, as well as microbiota transplantation.

Moreover, hypertension and insulin resistance caused by obesity, and hyperinsulinemia, the central factor of metabolic syndrome, drive glomerular hyperfiltration, albuminuria, endothelial dysfunction, and oxidative stress, all contributing to the progression of kidney damage [5, 27].

Although these mechanisms are involved in the pathogenesis of ORG, ORG does not occur in all obese individuals. Studies suggest that additional risk factors may contribute to susceptibility of ORG, including low nephron number due to low birth weight, prematurity, or congenital kidney anomalies; a history of prior kidney injury or nephrectomy; and the presence of visceral adiposity, nonalcoholic fatty liver disease and obstructive sleep apnea [27–29].

### Clinical features and diagnosis

Patients with O-FSGS commonly present with proteinuria as an initial symptom, kidney dysfunction may also be present. While most patients develop sub-nephrotic proteinuria (< 3.5 g/day), about 30% may progress to nephrotic-range proteinuria, but they rarely develop frank nephrotic syndrome with significant hypoalbuminemia and edema. Dyslipidemia (70–80%) and hypertension (50–75%) may also develop in ORG patients. Over time, patients may experience a gradual increase in urinary protein levels, leading to a decline in GFR. In long-term follow-up, some patients with O-FSGS advance to ESKD. In contrast, primary FSGS often manifests with an abrupt onset of heavy proteinuria, primarily in the nephrotic range, and is commonly associated with full nephrotic syndrome, including hypoalbuminemia and generalized edema [10].

Praga et al. studied the clinical and pathological characteristics of 15 patients with O-FSGS compared to 15 non-obese individuals with primary FSGS. At the time of biopsy, patients with O-FSGS had an average proteinuria of 3.1 ± 2 g per day. During follow-up, protein levels increased to the nephrotic range in 80% of cases; however, none of these patients developed classical features of nephrotic syndrome such as edema or hypoalbuminemia. the mean glomerular diameter in patients with O-FSGS was 256 ± 24 μm, significantly larger than the 199 ± 26 μm observed in primary FSGS patients (P < 0.001). 46% of patients with O-FSGS experienced progressive kidney insufficiency with 5 patients requiring dialysis; the study highlighted that baseline creatinine and creatinine clearance were a strong predictor of disease progression [30]. In another study of 57 patients with O-FSGS compared to 50 patients with primary FSGS, the O-FSGS group was older, had lower rates of nephrotic syndrome (7% vs. 54%), and showed milder proteinuria, higher serum albumin, and less edema. Glomerulomegaly was present in all O-FSGS cases but only in 10% of primary FSGS. O-FSGS also showed less mean percentage segmental sclerosis (12% vs. 39%) and less podocyte foot process fusion. Renal outcomes were better in O-FSGS, with lower rates of progression to ESKD (4% vs. 42%) [31].

Assessing kidney function in individuals with obesity presents challenges due to body size variations. Standard eGFR formulas adjust for BSA, but this adjustment can underestimate hyperfiltration [12]. Because the convention of indexing GFR to 1.73 m^2^ is based on outdated average body size, using it in patients with markedly higher BSA lowers the reported eGFR, even when actual filtration is preserved. This can lead to incorrect staging of kidney function, with obese patients appearing to have reduced GFR despite having normal or even elevated unindexed values [32].

### Diagnostic tools

#### Kidney function biomarkers

Sub-nephrotic proteinuria is the most common initial clinical finding in ORG. Traditional kidney function tests such as serum creatinine may underestimate dysfunction in obese individuals due to variations in muscle mass; Cystatin C is a more reliable marker of kidney function than serum creatinine, as it is less affected by age, gender, ethnicity, nutrition, or muscle mass. However, studies show it correlates with BMI, likely because of its link to fat mass. Using eGFR standard equations may lack accuracy in individuals with high BMI and measured GFR is often recommended when feasible. Among emerging biomarkers, neutrophil gelatinase-associated lipocalin (NGAL), kidney injury molecule-1 (KIM-1), and galectin-3 have shown promise in detecting early tubular and glomerular injury, even in patients with preserved GFR. Elevated urinary NGAL and galectin-3 have been associated with early signs of ORG and may help in predicting disease progression before conventional markers such as albuminuria become abnormal (Table 1) [13, 33]. In a study of 142 obese adolescents, several lipid metabolism and kidney damage biomarkers were evaluated for prediction of CKD. In those with elevated GFR, urine galectin-3 correlated with albuminuria and was an independent predictor of filtration changes (β = 0.35, p = 0.016). Among adolescents with normal GFR, higher urine NGAL (β = 0.28, p = 0.046) and galectin-3 (β = 0.31, p = 0.011), along with elevated serum cholesterol (β = 0.30, p = 0.022), were associated with obesity-related kidney damage. In the decreased GFR group, serum uric acid (β = 0.28, p = 0.035), urine NGAL (β = 0.31, p = 0.003), and daily megalin excretion (β = 0.34, p = 0.003) were the strongest predictors of kidney damage. These biomarkers predicted the development of CKD even in patients with normal or mildly reduced GFR. Urinary NGAL and galectin-3 reflected tubular damage and fibrotic changes, indicating their potential as early markers of ORG [34]. However, NGAL and galectin-3 are not disease-specific and can be elevated in various kidney or systemic inflammatory conditions. Therefore, their use in the context of ORG requires careful interpretation alongside clinical and histopathologic findings [35, 36]. Future research should focus on validating combinations of biomarkers that more precisely differentiate ORG from other glomerulopathies.

**Table 1:** Diagnostic modalities for ORG

Diagnostic tool	Category	Advantages	Disadvantages
Serum creatinine	Traditional kidney function test	Simple, widely used	Underestimates in obesity
Cystatin C	Biomarker	Reliable, Predictive	BMI-linked, Costly
eGFR standard equations	Kidney function test	Widely used, adjusts for BSA	Inaccurate with high BMI
Measured GFR	Kidney function test	Direct measurement	Requires specialized equipment
NGAL (neutrophil gelatinase-associated lipocalin)	Emerging biomarker	Early tubular injury detection	Not ORG-specific
KIM-1 (kidney injury molecule-1)	Emerging biomarker	Early tubular injury detection	Not ORG-specific
Galectin-3	Emerging biomarker	Predicts progression	Not ORG-specific
Proton magnetic resonance spectroscopy (^1^H-MRS)	Non-invasive imaging	Quantifies fat	Expensive
Kidney ultrasonography	Non-invasive imaging	Accessible, cost-effective, assessing kidney size, parenchymal changes, and vascular alterations	Limited fibrosis assessment
Ultrasound elastography	Non-invasive imaging	Detects fibrosis (non-invasive)	Expensive, limited
CT and MRI imaging	Imaging	Quantifies fat	Misses subtle changes
Kidney biopsy	Invasive diagnostic test	Gold standard	Invasive

#### Imaging

Ectopic lipid accumulation within the kidney, known as "fatty kidney," has emerged as a key factor contributing to ORG. Excessive fat deposition in kidney parenchymal cells (such as mesangial cells, podocytes, and tubular epithelial cells) can impair their normal function, leading to structural damage, and triggering inflammation and fibrosis. This lipid accumulation is closely associated with metabolically unhealthy obesity and may exacerbate glomerular hyperfiltration and albuminuria (through activating inflammatory pathways), driving disease progression. Recent advancements in non-invasive metabolic imaging, particularly proton magnetic resonance spectroscopy (^1^H-MRS), offer a promising tool to visualize and quantify ectopic fat in the kidney, providing new opportunities for identifying individuals at higher risk of ORG [37].

Kidney ultrasonography and ultrasound elastography are commonly used (Table 1). Color Doppler imaging can evaluate intrarenal blood flow and resistance indices, providing early signs of vascular damage. Ultrasound elastography offers non-invasive measurement of tissue stiffness (identifying early fibrosis). Computed tomography (CT) and magnetic resonance imaging (MRI) are also utilized to quantify ectopic fat accumulation, particularly renal sinus fat, which has been associated with impaired kidney function independent of overall obesity [29, 38].

#### Kidney biopsy

Kidney biopsy plays an important role in diagnosing ORG (including O-FSGS), predominantly to rule out other renal pathologies with similar clinical presentations and different management implications. Histologically, the hallmark finding of ORG is glomerulomegaly, characterized by enlarged glomeruli resulting from hyperfiltration-driven hypertrophy. Biopsy often reveals focal segmental glomerulosclerosis lesions, predominantly affecting the perihilar region of hypertrophic glomeruli. This pattern differs from primary FSGS, as fewer glomeruli are involved, indicating a slower disease progression. Additionally, segmental and mild podocyte foot process effacement is observed, in contrast to the diffuse effacement typical of primary FSGS. Tubulointerstitial fibrosis, tubular atrophy, and arteriosclerosis may also be present but typically less pronounced than seen with diabetic nephropathy or other etiologies of arteriosclerosis.

Immunofluorescence is negative other than non-specific IgM and C3 deposition in areas of sclerosis. In some cases, lipid depositions are found within podocytes, mesangial, and proximal tubular epithelial cells, reflecting ectopic fat accumulation. Biopsy is most helpful to rule out coexisting or alternative kidney diseases, which may require specific therapeutic approaches such as IgA nephropathy or other immune complex glomerulonephritis. Many obese patients with kidney dysfunction may have overlapping conditions like diabetic nephropathy or arterio-nephrosclerosis [29, 39]. Diabetic changes, such as focal mesangial sclerosis and thickening of the glomerular and tubular basement membranes, are commonly observed, even in non-diabetic individuals, suggesting overlapping pathways with diabetic kidney disease [39]. Therefore, kidney biopsy is recommended particularly when patients have eGFR < 30 ml/min/1.73 m.^2^, nephrotic syndrome or acute kidney injury [40].

A case series from China analyzed 90 patients with biopsy-proven ORG over five years. Histological findings revealed universal glomerulomegaly and FSGS in 70% of cases, with minimal tubular injury and preserved kidney function in most patients. Higher BMI correlated with increased proteinuria, glomerular hypertrophy, and greater foot-process width [41].

Of note, there is no consensus regarding the definition of glomerulomegaly on biopsy assessment. Many pathologists use an arbitrary cut off of glomerular diameters of > 50% of a single high magnification 40 × field. Mild enlargement may be overlooked or ignored and is likely under reported. Further work establishing a unified criteria for glomerular hypertrophy would be useful for future studies and aide in recognition of this pathologic change. Computational tools evaluating glomerular volume may be necessary to derive the most meaningful glomerular size data. Moreover, location of enlarged glomeruli (e.g., superficial versus deep cortex) may also be important information for diagnosis and prognostication [42, 43].

#### Management and treatment approaches

Treatment strategies for ORG primarily aim to reduce proteinuria to preserve kidney function. The most studied approaches include RAAS blockade, SGLT2 inhibitors, and weight-loss interventions through diet, pharmacotherapy, or bariatric surgery [44]. Management of comorbidities such as hypertension, Type 2 DM and dyslipidemia is cornerstone in reducing kidney injury in patients with ORG. For instance, blood-pressure control in patients with chronic CKD reduces incident albuminuria and slows decline in glomerular filtration rate. Moreover, glycaemic and lipid-profile management are similarly associated with better kidney outcomes [45, 46].

#### Lifestyle changes

Wight loss achieved through dietary intervention and regular exercise has been shown to play a significant role in improving clinical outcomes [19]. A study evaluated 38 overweight/obese patients with CKD stage 1–3 who underwent an 8-week lifestyle intervention (diet combined with exercise). Those who achieved the target body weight reduction of ≥ 3% from baseline had significant improvements in blood pressure control, serum creatinine (1.1 ± 0.3 vs. 0.8 ± 0.3; P < 0.001), proteinuria (76.3% vs. 50.0%; P = 0.02), and eGFR (75.9 ± 21.2 vs. 104.9 ± 38.1; P < 0.001) [47]. While lifestyle changes in treatment of ORG are beneficial, maintaining and adhering to them over the long-term remains challenging. This necessitates considering other alternative approaches for weight loss.

#### RAAS inhibitors and MR blockade

RAAS inhibition and mineralocorticoid receptor (MR) blockade are promising strategies in managing O-FSGS by reducing inflammation, proteinuria, and fibrosis. Targeting MR activation helps attenuate tubulointerstitial fibrosis and may slow disease progression (Table 2) [48]. Mallamaci et al. evaluated the efficacy of ramipril in chronic proteinuria they included 337 CKD patients with known BMI (31.1% were overweight and 14.5% were obese). The study evaluated the impact of obesity on kidney outcomes (ESKD and combined outcome of ESKD or doubling of serum creatinine) and assessed the response to ramipril compared to placebo. Obese patients in the placebo group had the highest rate of ESKD, while treatment with ramipril significantly reduced these risks across all BMI groups. The greatest benefit was observed in obese patients, with an 86% reduction in ESKD incidence and a 79% reduction in the combined endpoint [49].

**Table 2:** Current therapies and supportive care for ORG

Treatment type	Mechanism of action
RAAS inhibition	Reduces inflammation, proteinuria, and fibrosis by blocking RAAS and MR activation
SGLT2 inhibitors	Reduces hyperfiltration, lowers blood pressure, and reduces inflammation by inhibiting SGLT2
GLP-1 receptor agonists	Reduces glomerular hyperfiltration, inflammation, and oxidative stress while aiding in weight loss and insulin sensitivity
Bariatric surgery	Improves kidney function by reducing albuminuria, eGFR decline, and glomerular hyperfiltration

*RAAS* Renin–Angiotensin–Aldosterone System, *MR* Mineralocorticoid Receptor, *SGLT2* Sodium–Glucose Cotransporter 2, *GLP-1* Glucagon-Like Peptide 1 *eGFR* Estimated Glomerular Filtration Rate

Morales et al. studied the added renoprotective effect of aldosterone antagonists in combination with RAAS blockers in obese patients with proteinuria. They analyzed 71 patients with significant proteinuria (> 1 g/24 h), including 32 obese (BMI ≥ 30 kg/m^2^) and 39 control individuals, all receiving spironolactone alongside other RAAS inhibitors. After a median follow-up of 28.9 months, proteinuria decreased significantly in both groups. [50].

#### SGLT2 inhibitors

SGLT2 inhibitors such as empagliflozin and dapagliflozin are promising therapeutic agents for ORG because of their diverse benefits on the kidney (in addition to their glucose-lowering effects). They reduce intraglomerular pressure by promoting natriuresis and decreasing hyperfiltration. SGLT2 inhibitors contribute to improved renal outcomes by decreasing hemodynamic stress and reducing inflammation and fibrosis in the kidney [22]. A systematic review and meta-analysis showed SGLT2 inhibitors were linked to a reduced risk of CKD progression in patients previously diagnosed with CKD, with a relative risk of 0.77 (95% CI 0.68–0.88) compared to placebo [51]. A study investigated the renoprotective effects of empagliflozin (EMPA) in mice with ORG induced by a high-fat diet. Using multi-tissue metabolomic and gut microbiota analyses, data showed EMPA significantly improved kidney function and reduced glomerular damage, lipid accumulation, and inflammation. These effects were linked to the modulation of lipid metabolism, particularly glycerophospholipid metabolism and the pantothenate and CoA biosynthesis pathway, through alteration in the gut microbiota [52].

#### GLP-1 receptor agonists

GLP-1RAs are increasingly being used in the treatment of type 2 DM. They mimic the action of endogenous GLP-1 hormone -an Incretin hormone that is secreted by the small intestine in response to food intake [53]. Recent studies demonstrated GLP-1RAs benefits in weight loss and cardiovascular risk reduction [53, 54]. GLP-1RAs also have demonstrated significant kidney protective effects in addition to their glucose-lowering and weight loss effects. GLP-1RAs also suppress pro-inflammatory and profibrotic pathways, thereby minimizing oxidative stress and protecting against ischemic injury [55]. GLP1RAs can reduce glomerular hyperfiltration. Furthermore, GLP1RAs reduce the progression or onset of macroalbuminuria in individuals with type 2 diabetes [56]. In patients with CKD and overweight or obesity, GLP-1RAs (especially semaglutide) have shown beneficial kidney effects, including a reduction in albuminuria and a slower decline in eGFR. In the FLOW trial, semaglutide caused a 24% relative risk reduction in the composite outcome of kidney failure, eGFR decline ≥ 50%, or kidney-related death (HR 0.76; 95% CI: 0.66–0.88) [57, 58].Despite their benefits, they have some limitations such as high cost and drug adverse effects that sometimes lead to drug discontinuation. Gastrointestinal disturbances such as nausea, vomiting, diarrhea, and constipation are the most reported adverse effects, and are often dose-dependent [59].

#### Bariatric surgery

A variety of bariatric procedures, including sleeve gastrectomy and Roux-en-Y gastric bypass, have demonstrated kidney protective effects in ORG by improving albuminuria and eGFR, reducing glomerular hyperfiltration, and attenuating CKD progression [60]. A systematic review and meta-analysis on bariatric surgery in obese patients with impaired kidney function showed reductions in glomerular hyperfiltration (RR: 0.46, 95% CI 0.26–0.82), albuminuria (RR: 0.42, 95% CI: 0.36–0.50), and proteinuria (RR: 0.31, 95% CI: 0.22–0.43), after bariatric surgery, in addition to a notable decrease in measured GFR (SMD: −1.62, 95% CI: −2.63–0.60), reflecting improvement in hyperfiltration [61]. In a retrospective study of 2247 obese patients, bariatric surgery caused significant improvement in kidney function over a 3-year follow-up. The urine-albumin-creatinine ratio (UACR) decreased from 40.3 to 11.1 mg/g, and mean eGFR increased from 79.4 to 87.3 mL/min. The prevalence of microalbuminuria dropped from 13.2% to 6.3%, macroalbuminuria declined from 2.5% to 0%, and hyperfiltration reduced from 4.4% to 2.7% [62].

In conclusion, ORG, characterized by glomerulomegaly with or without FSGS, is a growing complication of obesity worldwide. An integrated approach combining lifestyle modification, pharmacotherapy, and bariatric surgery proves effective in ORG treatment. However, there is a need for clinical trials to evaluate targeted therapies in these patients.

## Data Availability

No new datasets were generated or analyzed. All data discussed in this article are available in the published literature cited within the manuscript.
